# Translational inhalable extracellular vesicle‐based mRNA therapy for the treatment of lung cancer

**DOI:** 10.1002/ctm2.70186

**Published:** 2025-01-12

**Authors:** Mengrui Liu, Brian Henick, Ke Cheng

**Affiliations:** ^1^ Department of Biomedical Engineering Columbia University New York New York USA; ^2^ Herbert Irving Comprehensive Cancer Center Columbia University New York New York USA

Lung cancer remains the leading carcinoma type in morbidity and mortality, distinguished by one of the lowest five‐year survival rates. Despite improvements with incorporation of immune checkpoint therapy in certain contexts, systemic immune‐related adverse events and the immunosuppressive tumour microenvironment(TME) constrain their effectiveness. Consequently, cytokines have emerged as a next‐generation immunotherapy that can convert immunologically ‘cold’ tumours into ‘hot’ ones.^1^ Interleukin‐12 (IL12), a potent cytokine, has demonstrated significant efficacy in stimulating interferon‐γ (IFN‐γ) production and enhancing the cytolytic activity of immune cells. However, current research on IL12 normally focuses on intratumour injection due to the off‐target‐induced toxicity in systemic delivery.^2^ Therefore, developing targeted and localised delivery platforms for IL12 is critical for deep‐organ tumours, especially lung cancer. Our research introduces IL12 messenger RNA (mRNA)‐loaded exosomes (IL12‐Exo), which are directly delivered into the lung TME through inhalation, providing a groundbreaking approach to lung cancer immunotherapy.^3^

## 1 TRANSLATIONAL EXOSOME‐BASED DELIVERY PLATFORMS

The efficacy and safety of IL12 therapy hinge on its successful local delivery, which depends on enhanced targeting to the lung tumours and minimised leakage into the blood circulation and off‐target organs. The mRNA of IL12 serves as an ideal delivery cargo, with its intratumoural delivery already advancing to clinical trials since the mRNA ensures local translation.^4^ Additionally, extracellular vesicles, especially exosomes, are natural vesicles derived from cells and offer tremendous therapeutic potential through their inherent cargos or external mRNA delivery platforms.^5^, ^6^, ^7^ Notably, we have harnessed lung spheroid cells as a novel source of therapeutic exosomes,^8^, ^9^, ^10^ now in a first‐in‐man clinical trial for patients with idiopathic pulmonary fibrosis. We have also innovated various exosome‐mediated delivery systems for lung‐specific diseases, including COVID‐19,^7^, ^11^, ^12^ demonstrating the effectiveness of inhalation as a non‐invasive and localised administration route.^8^, ^13^, ^14^, ^15^, ^16^, ^17^, ^18^, ^19^

**FIGURE 1:**
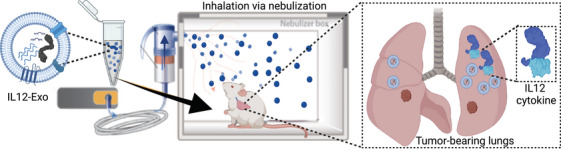
Schematic illustration of inhalation delivery of IL12‐Exo. IL12‐Exo was administered by nebulised inhalation to the lungs of tumour‐bearing mice, leading to localised expression of IL12 cytokine in the lung TME. Tumour cells were intravenously injected into mice to establish lung tumour and metastasis models. The figure was created with BioRender.com.

Upon nebulised inhalation in a mouse model with lung tumours, IL12‐Exo achieved targeted delivery and localised expression of IL12 cytokine (Figure 1). Neutralised inhalation delivery of IL12‐Exo showed superior accumulation in the lung compared to other off‐target organs, and the exosome platform exhibited a specific affinity for tumour cells over the IL12 mRNA‐loaded liposome (IL12‐Lipo) control. These findings align with our previous research indicating superior delivery of mRNA and protein in healthy lungs when encapsulated in exosomes rather than liposomes.^20^ As expected, the enhanced localisation led to sustained IL12 protein encoding, significantly reducing its systemic leakage and addressing the primary concern of IL12‐related toxicity. Importantly, IL12‐Exo therapy induced robust IFN‐γ production in the lung TME, a crucial therapeutic mechanism of IL12 treatment.

## 2 STIMULATION OF ANTI‐TUMOUR IMMUNE RESPONSES

**FIGURE 2:**
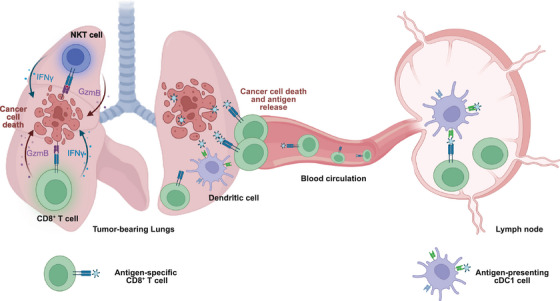
Schematic illustration of the immune response in the lung TME and systemic T cell protection post‐IL12 therapy. IL12‐Exo treatment primarily induced IFN‐γ expression in CD8^+^ T cells, with some expression also observed in natural killer T (NKT) cells. Granzyme B expression was induced in these two cell types, equipping them with direct tumour‐killing ability. Additionally, IL12‐Exo therapy activated systemic T cell protection. In specific, it enhanced the expression of cDC1 cells, followed by antigen priming in the lymph nodes, leading to tumour‐specific antigen expression on peripheral CD8^+^ T cells. The figure was created with BioRender.com.

Localised IL12 expression and IFN‐γ production mediated by our delivery system facilitated robust immunotherapy, including direct tumour eradication, enhanced infiltration of anti‐tumour immune cells in the lung TME, and promotion of systemic immunity and immune memory. For the mechanism, CD8^+^ T cells were demonstrated as the predominant effector cells, showing increased and responsive IFN‐γ production post‐IL12 treatment. Further, IL12 therapy fostered systemic T‐cell protection, where the induction of CD8^+^ T cells depended on the activity of cross‐presenting type 1 dendritic cells (cDC1 cells). This was evidenced by activated cDC1 cells in lymph nodes and the specific production of tumour antigen‐specific T cells in the circulation (Figure 2). This primed T cell response conferred resistance to long‐term survival rates against lung tumour and metastasis and even tumour rechallenge.

## 3 ROBUST IMMUNOTHERAPY WITH A SIMPLIFIED APPROACH

This strategy exemplifies a potent yet straightforward IL12 mRNA delivery system to the lung TME. The preparation process is streamlined without the requirement of chemical reactions, which simplifies manufacturing and ensures a high level of purity and biocompatibility, crucial for patient safety. Notably, inhalable exosomes provide a non‐invasive, patient‐centred method for delivering IL‐12 mRNA directly to lung cancer sites. This approach avoids the discomfort and systemic side effects associated with conventional intravenous therapies. By allowing direct administration into the lungs, it maximises therapeutic concentration at the lung TME and minimises systemic exposure. Additionally, the potential for home administration enhances patient convenience, significantly reducing both logistical and financial burdens.

## 4 CLINICAL PERSPECTIVE AND FUTURE OUTLOOK

This localised administration therapy could revolutionise treatment for patients with primary and acquired resistance to approved immunotherapies and offer a safer treatment modality for populations where systemic immunotherapy poses risks, such as transplant recipients and those affected by autoimmune disease. Beyond treating refractory non‐small cell lung cancer, this method could also benefit patients with cancers from other organs metastatic to the lungs. Window‐of‐opportunity studies are feasible for these patient groups with resectable disease, in whom standard‐of‐care neoadjuvant chemoimmunotherapy cannot be safely administered. The successful development and validation of IL12‐Exo promise to usher in a new era of immunotherapy against lung cancer, utilising inhalable exosome‐mediated gene nanomedicines.

## AUTHOR CONTRIBUTIONS

M.L. prepared the initial draft of the manuscript. M.L., B.H., and K.C. contributed to the revision of the manuscript. All authors reviewed and approved the final version. All authors provided written permission to the corresponding author to be acknowledged in the manuscript.

## CONFLICT OF INTEREST STATEMENT

BSH: Consultant to Genentech‐Roche, Jazz Pharmaceuticals, AstraZeneca, Regeneron, Bristol‐Myers Squibb, Boehringer Ingelheim, Bayer, Synthekine. Grants/contracts to institution: Janssen, Genentech‐Roche.

## FUNDING INFORMATION

This work is supported by grants from the National Institute of Health (NIH) of the USA (HL123920, HL137093, HL144002, HL146153, HL147357, HL 149940 to K.C.), and partly funded through the NIH/NCI Cancer Center Support Grant P30CA013696.
